# Magnocellular Vasopressin and the Mechanism of “Glucocorticoid Escape”

**DOI:** 10.3389/fendo.2019.00422

**Published:** 2019-06-26

**Authors:** Ferenc A. Antoni

**Affiliations:** Centre for Discovery Brain Sciences, Deanery of Biomedical Sciences, University of Edinburgh, Edinburgh, United Kingdom

**Keywords:** vasopressin, adrenal corticosteroids, ACTH, dexamethasone non-suppression, interleukin-6, agonist-induced plasticity

## Abstract

It is now widely accepted that magnocellular vasopressinergic neurons in the supraoptic and paraventricular nuclei participate in the control of adrenocorticotropin secretion by the anterior pituitary gland. However, it remains to be explored in further detail, when and how these multifunctional neurons are involved in the control of anterior pituitary function. This paper highlights the role of magnocellular vasopressin in the hypothalamic pituitary adrenocortical axis with special reference to escape from glucocorticoid feedback inhibition. The signaling mechanisms underlying glucocorticoid escape by pituitary corticotrope cells, as well as the wider physiologic and pathologic contexts in which escape is known to occur—namely strenuous exercise, and autoimmune inflammation will be considered. It is proposed that by inducing escape from glucocorticoid feedback inhibition at the pituitary level, magnocellular vasopressin is critically important for the anti-inflammatory, and immunosuppressant actions of endogenous corticosteroids.

## Introduction

This paper is the consequence of a symposium held in the honor of the late Dr. Kevin J. Catt (1932–2017) at Semmelweis University in Budapest. As a first year student at this institution, which has a tradition of teaching medicine for 250 years, I was handed the course textbook, “Functional Anatomy” by János Szentágothai (Schimmer) (1). First published as a single author book in 1971, this tome managed a readable, if in places decidedly subjective synthesis of classic gross anatomy and histology with modern studies of ultrastructure, physiology, and biochemistry. The didactic approach was unashamedly teleologic in emphasizing the unity of form and function. However, it went beyond Aristotelian metaphysics. It was an almost poetic celebration of the harmony of the human body, of how, from single cells to complex organs, structure reflected purpose, and function.

The scientific oeuvre of Kevin Catt heralds the beginning of a new era where the structures and regulatory properties of cells are pared back to their ultimate molecular constituents. Now in full-swing, this era of discovery is best symbolized by the complex analysis of individual cells, involving the combination of multi-parameter imaging with transcriptomics, and proteomics (2). We are able to map how the seemingly stochastic expression of genes underpins the functional plasticity of cell populations to support macroscopic physiologic reflexes and control mechanisms. This review aims to follow the path of Szentágothai in the quest for achieving the synthesis of structure and function, also incorporating the molecular insights of cellular signaling pathways pioneered by Kevin Catt.

## An Outline of the Hypothalamic-Pituitary-Adrenocortical Axis

The hypothalamic-pituitary-adrenocortical system comprises a classic neuroendocrine regulatory feedback circuit. Neuroendocrine neurons in the hypothalamus drive the secretion of adrenocorticotropin (ACTH) by releasing neuropeptides, namely 41-residue corticotropin releasing-factor (CRF) and vasopressin (VP), into hypophysial portal blood-vessels that irrigate the anterior pituitary gland (3). These neurons receive a large number of diverse neural inputs from various parts of the brain that contribute to the hormonal response to stress (4–8). The production and secretion of steroid hormones—glucocorticoids, as well as mineralocorticoids—by the adrenal cortex is stimulated by ACTH secreted by the pituitary gland. An important effect of the steroids is to provide inhibitory feedback at the level of hypothalamic neroendocrine neurons, adenohypophysial corticotropes, as well as the higher brain centers involved in the control of neuroendocrine neurons (9, 10). The relative importance of the site(s) of feedback inhibition that govern the size and duration of the HPA response to stress may be dependent on the biological paradigm analyzed. In clinical practice, HPA responsiveness is usually assessed by some form of the dexamethasone suppression-test (11), utilizing a synthetic steroid largely selective for Type II glucocorticoid receptors (10). The main site of feedback tested by this paradigm appears to be the anterior pituitary corticotrope cell (12, 13).

## Vasopressin

Vasopressin (VP) is a small, COOH-terminally amidated peptide chiefly synthesized by nerve cells, hence designated as a neuropeptide. Arginine-vasopressin is the most common form in mammals, but in some species Arg is replaced by Lys see review by Bichet (14). Vasopressin is elaborated upon the processing of a larger protein precursor—preprovasopressin (15), which is packaged and processed in dense-core secretory granules that are transported to sites of release along the axon as well as the dendrites (15, 16). Note that neurophysin II and copeptin, the COOH-terminal segment of the precursor, are also co-secreted with VP. The biological role of copeptin is unknown. However, its stability in human plasma and immunogenicity has been exploited to use copeptin as surrogate for the activity of VP-secreting neurons with intriguing results (17, 18). The cellular actions of VP are mediated by G protein-coupled 7-transmembrane domain receptors. Of these, the V1b isoform appears most relevant for the stress response (19, 20).

## Functional Neuroanatomy of Vasopressinergic Neurons of the Hypothalamus

### The Magnocellular System

The magnocellular neurosecretory system of the hypothalamus was discovered a relatively long time ago, on the basis of the distinct histochemical reaction produced by the high amounts of neurohormone elaborated by these neurons reviewed by Scharrer (21). The cell bodies are found in the supraoptic and paraventricular hypothalamic nuclei and send their axons to terminate in the neurohemal interface zone located at the bottom of the hypothalamus. The neurohemal interface is comprised of the median eminence and the posterior lobe of the pituitary gland also referred to as the neurohypophysis. Magnocellular neurons constitute the classic antidiuretic system that is activated by hypernatremia or hypovolemia and releases VP into the systemic circulation (14). Subsequent work has shown that the axons of magnocellular neurons also release VP *en-passant* in the internal zone of the median eminence (22–24). The VP released by this process appears in pituitary portal blood that irrigates the anterior pituitary gland [(25), also see Box 1]. It is of note, that magnocellular VP neurons also release vasopressin from their dendrites (16). Whether or not dendritic release contributes to the control of the pituitary secretion of ACTH remains to be clarified. However, it is well-established that magnocellular neurons make various forms of contact with CRF producing cells in the medial parvicellular paraventricular nucleus of the hypothalamus (26, 27). Moreover, Plotsky and co-workers found that in rats VP given into the 3rd cerebral ventricle attenuated the release of CRF into hypophysial portal blood and, conversely, inhibition of AVP action increased the levels of CRF (28). These results indicate that there may be a functional link between the magnocellular VP neurons and parvicellular neurons producing CRF.

Box 1 **Six decades of dogma-busting: The magnocellular vasopressinergic neuron as a modulator of anterior pituitary ACTH secretion.**

1. Neurosecretion into the bloodstream-non-conforming to neuron doctrine.
2. Negates single hypothalamic releasing hormone for each pituitary hormone hypothesis.
3. VP is not just the antidiuretic hormone (ADH).
4. Secretory path from internal zone of the median eminence into pituitary portal blood-“*en passant*” neuropeptide release.
5. Negates the hyophyseotrophic area = the mediobasal hypothalamus concept.
6. VP alone has no ACTH releasing effect at physiologic concentrations i.e., it is a new type of neuroendocrine modulator, a major action of which is to induce glucocorticoid resistance at the level of the corticotrope cell.

### Parvicellular Neurons Producing CRF and VP

A second population of VP-ergic neurons projecting to the median eminence is located in the medial parvicellular part of the hypothalamic paraventricular nucleus. These are small neurons that also co-produce and co-package CRF with VP (29, 30) and are the key neuroendocrine neurons that regulate the pituitary secretion of ACTH (31, 32). With respect to the stress response two functional differences between the magnocellular and parvicellular neurons are of note. First, under normal conditions, magnocellular neurons produce up to 10-fold more VP than parvicellular neurons (30, 33). Second, the production and release of VP by parvicellular neurons is under prominent inhibitory control by adrenal corticosteroids (34), while that in magnocellular neurons is not (35).This is despite the fact that magnocellular VP neurons express Type II glucocorticoid receptors (36). In the absence of adrenal corticosteroids, VP release from the parvicellular neurons is massively increased and becomes comparable to that from magnocellular neurons (33, 37).

### The Relevance of Magno- and Parvi-Cellular VP-Ergic Neurons to the Stress Response

The role of VP and its pituitary V1b receptor in the stress response has been analyzed in considerable detail in various models, including genetically vasopressin-deficient Brattleboro rats (38) and V1b receptor gene-deleted mice (20). These studies, and many others see (39) for review, support an important role for VP in the stimulatory control of stress-induced ACTH secretion. However, very few studies have attempted to distinguish between the effects of parvicellular and magnocellular VP. Plotsky and Thivikraman first produced evidence showing that glucocorticoid negative feedback appeared to be absent in conscious rats subjected to repeated moderate hemorrhage (40). Subsequently, these workers showed that HPA activation by air-puff startle, a psychological stressor, was sensitive to inhibition by corticosterone whereas that caused by small or moderate hemorrhage was not (41). Moreover, they found early immediate gene activation in magnocellular neurons of the supraoptic and paraventricular hypothalamic nuclei during hemorrhage and activation in the dorsomedial part of the hypothalamus by air-puff startle. Thus, this work differentiated feedback prone and feedback resistant mild stressors and observed signs of activation in magnocellular hypothalamic neurons in the case of the latter.

## Signal Integration by Corticotrope Cells of the Anterior Pituitary Gland and the VP-Induced Escape From Inhibition by Glucocorticoids

The stimulatory input to corticotrope cells is comprised of CRF and VP. A hypothalamic ACTH-inhibiting factor, atrial natriuretic peptide (ANP) has also been identified (39, 42). Finally, corticotrope cells are a well-established site of glucocorticoid feedback-inhibition (9, 10).

### CRF

The widely accepted mechanism of action in the case of CRF is activation of CRF1 receptors and conservative coupling to the stimulatory G protein Gs. The consequent activation of transmembrane adenylyl cyclase(s) leads to increase of intracellular cAMP levels (43). The scenario of intracellular signaling downstream of cAMP is complex, as reviewed in detail elsewhere (44, 45). However, it is clear that intracellular Ca^2+^ signals are generated by CRF, and that the electrical activity of the cells ultimately determines the secretion of ACTH.

### VP

In the case of VP, the consensus is the activation of V_1b_ receptors coupled to Gq and consequent activation of PLC, with ensuing increases of IP3 levels and PKC activity. Exactly which isoform(s) of PKC is (are) involved, is not entirely clear, but as phorbol esters can mimic most of the known actions of VP including the potentiation of the cAMP signal and the stimulation of ACTH secretion, the alpha and beta PKC-isoforms are the likely candidates. Once more, events after IP3 and PKC activation are too complex to be considered here (44, 46).

### ANP

There is a signaling pathway to inhibit stimulated ACTH secretion through 3′5′guanosine monophosphate (cGMP), that is not shared with glucocorticoids (47). This pathway can be activated by ANP (48, 49), and may become functionally more prominent under conditions where the pituitary corticotropes are in “glucocorticoid escape” mode.

### Glucocorticoids

Feedback inhibition in the HPA axis occurs in three time-domains; rapid <15 min, early delayed—within 20 min and up to 2 h, and delayed, beyond 2 h (9, 10, 50). With respect to the time-domains of feedback manifested in corticotropes: in contrast to a recent study (51), we have failed to observe a rapid inhibitory effect of glucocorticoids in dispersed rat anterior pituitary (52) or AtT-20D16:16 mouse corticotrope tumor cells (53). Thus, the focus of the studies outlined here was early-delayed feedback, which is the main inhibitory effect in the first 2 h after exposure to glucocorticoids. The properties of this inhibition have been reviewed extensively (9, 54, 55). In brief, it is mediated by Type II glucocorticoid receptors and is sensitive to inhibitors of transcription and translation, indicating a requirement of mRNA synthesis/processing and protein synthesis (52). What is the molecular and cellular basis of early- delayed inhibition? In AtT-20 cells, the balance of cAMP-dependent phosphorylation on the stress-axis-regulated-insert (STREX) type α-subunit of the large conductance Ca^2+^-dependent K^+^-channel (BK-channel) is shifted toward dephosphorylation. As a result, the channel is not inhibited by cAMP-dependent phosphorylation induced by CRF (56). Hence, the enhancement of intracellular free Ca^2+^ transients and the consequent stimulation of ACTH secretion by CRF are suppressed, reviewed in refs (54, 57). Subsequent work has implicated a protein phosphatase 2A-like enzyme as the mediator of glucocorticoid action (58), but definitive evidence is still outstanding. In contrast to AtT20 cells, blockade of BK-channels had no appreciable effect on the efficacy of corticosterone to inhibit CRF-induced ACTH secretion in cultured rat anterior pituitary cells (59). This is despite the fact, that BK channels play a prominent role in governing the firing pattern and the intracellular Ca^2+^ transients of anterior pituitary cells in culture (57) and that corticosterone has an inhibitory effect on the bursting activity of mouse corticotropes cells (44). Moreover, inhibition of agonist-induced ACTH release by ANP/cGMP, could be reversed by blockers of BK as well as other K^+^ channels (60). In contrast, studies with a variety of K^+^ inhibitors failed to identify conclusively the K^+^ channel key for early delayed inhibition by corticosteroids (61). Others, using a similar panel of K^+^ channel blockers, reported a role for *ether-á-go-go*-related gene K^+^-channels as the mediators of glucocorticoid inhibition (62). However, these findings have not been confirmed by other, more direct methods, such as patch-clamp electrophysiology or gene-knock down. In summary, current evidence indicates that early-delayed corticosteroid feedback inhibition at the pituitary level is by glucocorticoid-induced protein(s) that facilitate the activation of K^+^-channels. A similar conclusion was reached in a study of hippocampal pyramidal neurons (63). Significantly, this scenario implies that glucocorticoid inhibition is sensitive to depolarization of the membrane potential (59, 64).

One of the complicating factors in understanding signaling processes in corticotropes is the variety of the *in vitro* models used. It is clear that cultured cells, acutely dispersed cells, and pituitary slices may have different properties. Moreover, the temporal dynamics in perifused tissue are different to that seen in static systems. Finally, the concentrations of the effectors employed tend to vary from physiologic (CRF up to 0.3 nM, VP up to 3 nM) to the grossly aphysiologic.

**Figure 1:**
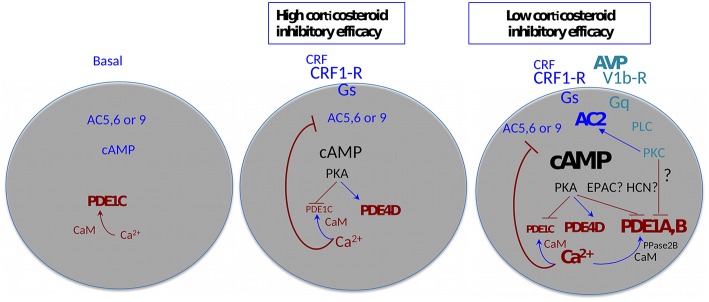
Agonist-induced plasticity of cAMP signaling—multiple functional states of the adenohypophysial corticotroph cell. AC, adenylyl cyclase; CaM, calmodulin; EPAC, exchange factors directly activated by cAMP; HCN, Hyperpolarization activated cyclic nucleotide gated channels; PDE- cyclic nucleotide phosphodiesterase; PKA, cAMP dependent protein kinase; PKC, protein kinase C; PLC, phospholipase C; PPase2B, protein phosphatase 2B (*alias* calcineurin). Arrows indicate facilitation, T-bars indicate inhibition. The size and thickness of the lettering is proportional to the amplitude/activity of the denoted entity.

With these considerations in mind, a detailed analysis of CRF-induced cAMP levels was carried out in the context of the inhibition of cAMP synthesis by intracellular free Ca^2+^, and the efficacy of corticosteroid feedback inhibition of stimulated ACTH release. It transpired that CRF-induced cAMP synthesis is under feedback inhibition by Ca^2+^ derived from a ryanodine/caffeine sensitive intracellular pool (65). However, above 1 nM CRF, the effect of Ca^2+^ was no longer apparent. A similar “escape” of CRF-induced cAMP synthesis from Ca^2+^ inhibition could be induced by physiologic concentrations of VP, an effect also mimicked by phorbol-ester activation of protein kinase C (65). Analysis of cAMP hydrolysis indicated that while PDE4 isoforms were active when low concentrations of CRF were used, inhibitors of PDE1 were required for inhibiting cAMP breakdown when CRF was applied in combination with VP. When interpreted in the context of the diversity of cAMP signaling proteins (66, 67), the results indicate that physiologic concentrations of CRF activate a Ca^2+^ inhibited adenylyl cyclase such as AC5, 6, or 9, while in the presence of VP an adenylyl cyclase activated by protein kinase C such as AC2 or AC7 enters the fray (Figure 1). This latter cyclase is responsible for the markedly increased cAMP levels that require high-capacity, low-affinity phosphodiesterases to control cAMP signaling. By the combination of immunocytochemistry, Western blotting and RT-PCR, we concluded that AC7, PDE1a, and PDE1b were the best candidates underlying the switching of corticotropes to high cAMP levels in the context of stimulation by CRF and VP. Importantly, VP was present at concentrations that are only produced by magnocellular neurons under physiologic conditions.

It is pertinent to highlight here that the “amplitude modulation” of the cAMP signal outlined above has potentially important consequences: whilst PKA is maximally active at 1 μM cAMP, other effectors such as EPACS, hyperpolarization-activated cyclic nucleotide–gated (HCN) channels, and cyclic nucleotide gated channels all require >10 μM cAMP for activity. Thus, a whole plethora of cAMP signaling systems is potentially mobilized in corticotrope cells in the presence of high (magnocellular) concentrations of VP that are not active at levels of stimulation that can be achieved *via* parvicellular CRF/VP. It is also important to reiterate here that the effects of VP on cAMP synthesis are conditional to the presence of CRF.

Several of the inferences in the aforementioned studies were made on the basis of the application of pharmacologic agents or the use of antibodies. It later transpired, that the antibodies used to localize AC2 in the anterior pituitary gland were unreliable, similarly, an antibody against AC5/6 gave also inconsistent results (68). More recently, mRNA-seq analyses of single anterior pituitary cells have been published (69). The data show selective enrichment of AC2 in corticotrope cells, in contrast, the levels of AC7 mRNA in anterior pituitary cells are very low. All three Ca^2+^-inhibited ACs, AC5, 6, and 9 appear to be expressed in corticotropes. Moreover, the highest relative levels of PDE1a and PDE1b appear to be associated with cells identified as corticotropes.

Taken together, the molecular machinery of Ca^2+^ feedback on cAMP biosynthesis and for achieving the VP-mediated boost of cellular cAMP to levels that address EPACS, HCNs and CNGs is present in corticotropes. Finally, is attractive to speculate here, that high VP overrides early feedback through depolarization of the membrane potential by activation of an HCN channel (70, 71).

The analysis of the efficacy of corticosterone to inhibit CRF-induced ACTH release showed interesting parallels with the characteristics of the inhibition of cAMP synthesis by Ca^2+^(61). In the physiologic range for CRF, corticosterone inhibition was highly efficient, while at supraphysiological CRF the inhibitory efficacy was markedly reduced. Application of the PDE4 blocker rolipram or high, physiologic levels of VP with physiologic concentrations of CRF also markedly reduced the inhibitory efficacy of corticosterone. In essence, irrespective of how intracellular cAMP levels were elevated above 20 μM, glucocorticoid efficacy was progressively reduced. Thus, if the agonists by-pass the Ca^2+^ inhibitory feedback on adenylyl cyclase, the efficacy of glucocorticoid inhibition is dramatically reduced. These observations link the VP mediated switch of low cAMP signaling induced by CRF to high cAMP, with the escape from glucocorticoid feedback at the pituitary level. A summary cartoon of the corticosteroid sensitive and insensitive states of anterior pituitary corticotrope cells is shown in Figure 1.

## When Is Glucocorticoid Escape of Biological Importance?

The pronounced deleterious effects of long-term corticosteroid excess on body homeostasis indicate that glucocorticoid escape is encountered in life-threatening situations and is likely to be of relatively short duration. The classic clinical examples of persistent glucocorticoid non-suppression are Cushing's disease (72) and melancholic depression (12).

### Autoimmune Inflammation

**Figure 2:**
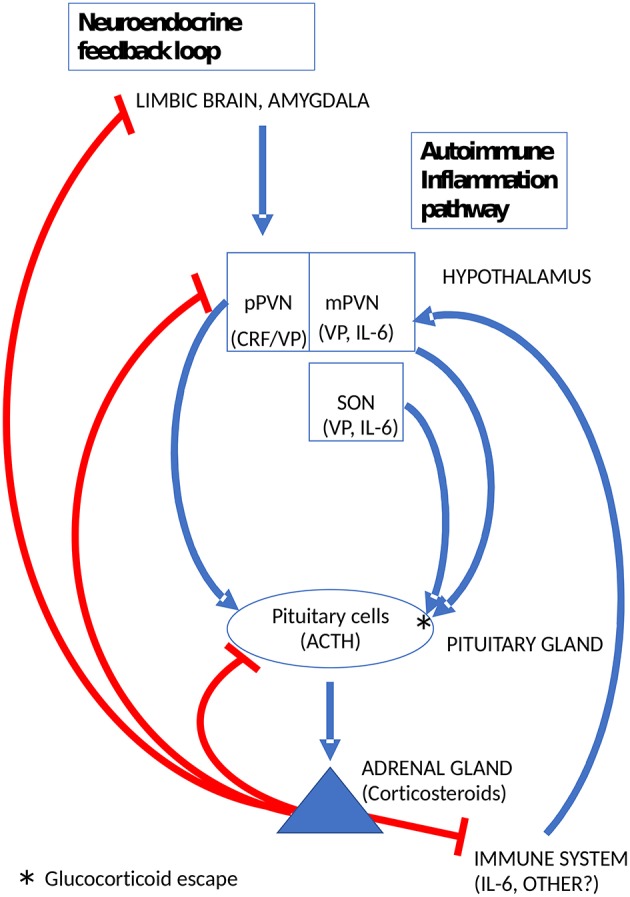
Glucocorticoid escape at the pituitary gland induced by magnocellular VP to mobilize the endogenous immunosuppressant activity of adrenal corticosteroids. pPVN, parvicellular medial dorsal part of the hypothalamic paraventricular nucleus; mPVN lateral magnocellular part of the PVN; SON, supraoptic hypothalamic nucleus.

In preclinical models of autoimmune inflammation, such as experimental autoimmune encephalomyelitis and adjuvant-induced arthritis, a sustained rise of plasma corticosteroids lasting several days is crucial for survival (73). Indeed, it is widely recognized that adrenal corticosteroids are important facilitators of endogenous immunosuppression (74). The only way this can occur is by escape from glucocorticoid feedback inhibition. Several lines of evidence indicate, that the sustained and vital increase of corticosteroids in preclinical autoimmune models is mediated by VP (75, 76). Moreover, the data of Suzuki etal. (76), indicated at the level of VP expression rather than mRNA, that magnocellular vasopressinergic neurons become activated during autoimmune inflammation. It is relevant to recall here that VP synthesis is not closely controlled by adrenal corticosteroids in magnocellular neurons (35) and that the effects of low levels of CRF on corticotropes are amplified dramatically by VP (65, 77). Thus, activation of magnocellular VP neurons can bypass glucocorticoid feedback in the CNS and induce escape from glucocorticoid feedback at the pituitary level to support the sustained secretion of corticosteroids. The trigger for the activation of hypothalamic neurons during autoimmune inflammation is unknown. It is plausibly a cytokine, above all, interleukin-6 (IL-6) (78) known to activate the HPA axis in humans as well as rodents (79). However, the exact mechanism by which this occurs remains to be clarified (80). Intriguingly, magnocellular VP neurons also produce IL-6 (81). Moreover, increases of IL-6 levels in magnocellular neurons were observed after various types of stress paradigms (82). Whether or not this IL-6 is involved in the regulation VP release e.g., as a paracrine mediator, is unknown. A summary diagram of the by-pass of glucocorticoid feedback in the HPA axis through the activation of magnocellular VP neurons by immune mediators is shown in Figure 2.

### High Intensity Exercise and Hyponatremia

Work by Deuster et al. (83) reported that high-intensity exercise results in dexamethasone non-suppression in men as well as women. One of the threats of high-intensity exercise is hyponatremia (84), which may be lethal due to the development of hypotonic encephalopathy (85). Increases of plasma VP are prominent under these conditions and it has been argued that exercise-associated hypernatremia is a case of inappropriate VP secretion (SIADH). It is likely, that IL-6 released from muscle cells during exercise is a trigger for VP secretion. Additionally, hyperhydration by the subjects during exercise may contribute to hyponatremia. In the pituitary portal system, where VP levels are considerably higher than in the periphery, the levels of vasopressin are sufficient to induce glucocorticoid escape at the pituitary level. The ensuing higher levels of ACTH would enhance aldosterone secretion by zona glomerulosa cells and improve sodium retention. In addition, VP has a direct action to stimulate aldosterone secretion in rat (86) as well as human zona glomerulosa cells (87). Thus, while exercise may symptomatically mimic SIADH in that plasma levels of VP increase despite hypoosmolarity, it may be a fluid-volume preservation reflex that simultaneously promotes sodium retention by bringing about glucocorticoid non-suppression at the pituitary level.

## Summary

In a day-to-day setting, the activity of the HPA axis is under powerful feedback inhibitory control. The situation during certain types of stress may be different. In a seminal review, Munck et al. (88) proposed that the physiologic rationale of stress-induced glucocorticoid production was to dampen and reset the production of powerful immune mediators induced by the stress response. Three decades, two dozen cytokines and a few microbiomes later, this concept is stronger than ever. In particular, the emergence of IL-6 as a pleiotropic stress-responsive protein and its activation of the hypothalamic CRF and VP systems is of particular note. If corticosteroids are controlled by strict neuroendocrine feedback tailored for stress-free and/or casual mild stress conditions, they could not be deployed to combat stress with a substantial inflammatory component. Increasingly, it appears that most types of stress also mobilize inflammatory mediators (89, 90). Similarly, given the threat of hyponatremia in severe hypovolemia, overriding inhibitory feedback to allow sodium retention through aldosterone may be key to survival. Therefore, feedback inhibition has to be plastic. Indeed, the functional neuroanatomy, as well as the molecular and cellular elements underpinning the plasticity of corticosteroid feedback inhibition are clearly apparent in the hypothalamo-hypophysial system. Where sustained secretion of adrenal corticosteroids is vital for survival, the evidence points to a critical role of magnocellular VP to induce glucocorticoid escape at the pituitary level. Whether or not this insight can be exploited therapeutically remains to be explored.

## Author Contributions

The author confirms being the sole contributor of this work and has approved it for publication.

### Conflict of Interest Statement

The author declares that the research was conducted in the absence of any commercial or financial relationships that could be construed as a potential conflict of interest.
